# Supplementary material for: Mutant APC reshapes Wnt signaling plasma membrane nanodomains by altering cholesterol levels via oncogenic β-catenin
Source: Nat Commun. 2023 Jul 19;14:4342. doi: 10.1038/s41467-023-39640-w (PMC10356786; doi:10.1038/s41467-023-39640-w)
Supplement: Supplementary file 1 — Supplementary Information [file 41467_2023_39640_MOESM1_ESM.pdf]

Supplementary Figures and Tables

Supplementary Figure 1

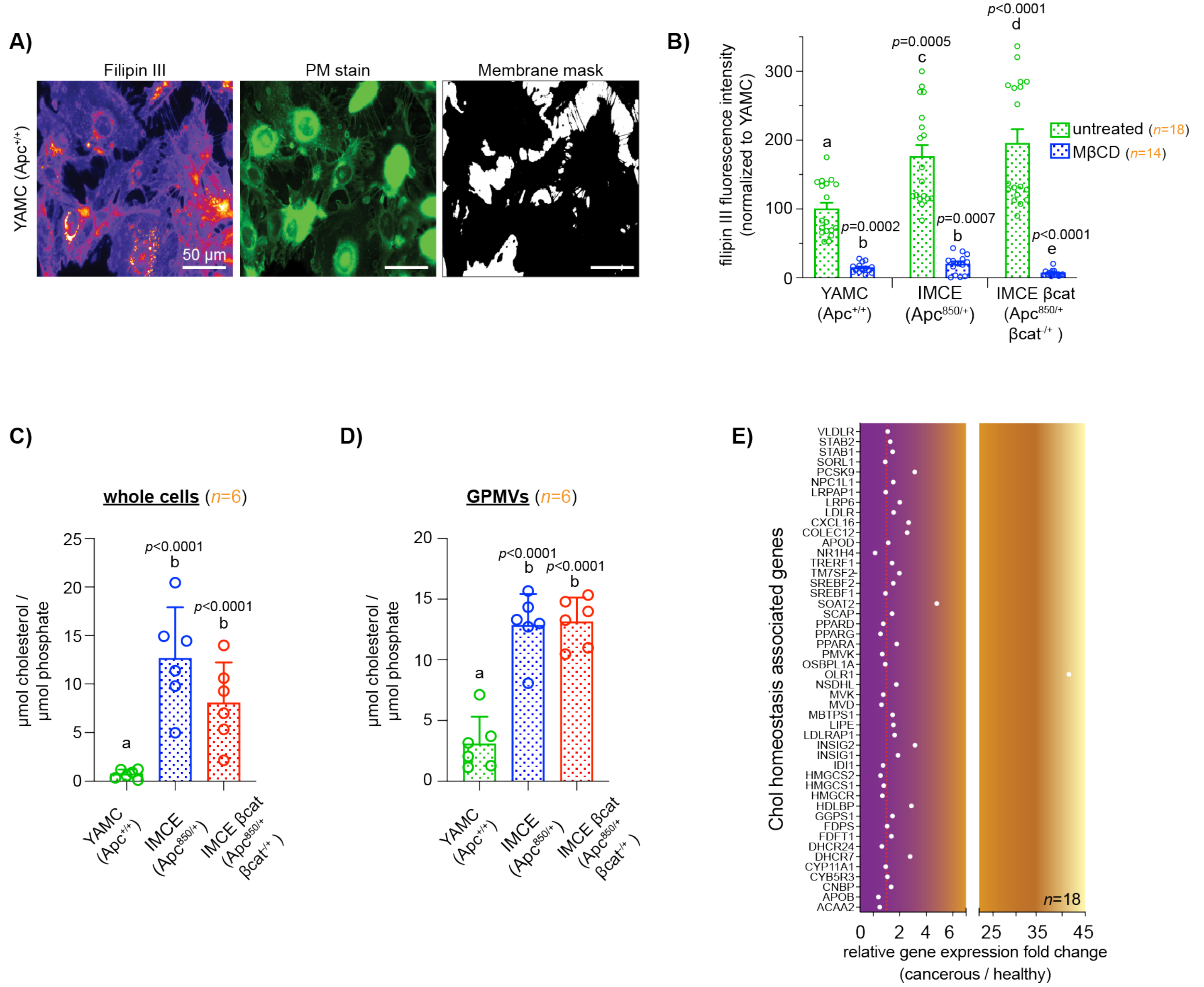

Figure S1. Oncogenic APC alters plasma membrane cholesterol homeostasis *in cellulo*. Fixed YAMC (Apc +/+), IMCE (Apc 850/+) and IMCE βcat (Apc 850/+ βcat -/+) cultured colonocytes were incubated with filipin III (cholesterol) (50 μg/mL) for 45 min in the dark, washed and imaged. As a control, select cultures were treated with methyl-β-cyclodextrin (MβCD) (10 mM) for 30 min prior fixation and filipin III incubation, as indicated. **A)** Representative images of YAMC cells stained with filipin III and CellMask™ DeepRed plasma membrane stain (5 μg/mL). Using the plasma membrane stain image, a membrane mask or region of interest (ROI) was generated in order to quantify filipin III intensity. Scale bars: 50 μm. **B)** Quantitative analysis of cholesterol levels in mouse colonic cell lines. Error bars represent filipin III fluorescence obtained from *n*=14-18 field of views (FOV) containing 5-10 cells per FOV (mean ± SD), normalized to untreated WT APC. Quantitative analysis of cholesterol levels in **C)** mouse colonocytes and **D)** their derived GPMVs. Cholesterol from whole colonocytes and GPMVs was extracted and quantified using an Amplex™ Red Cholesterol Assay kit. Total cholesterol was calculated from luciferase luminescence data and normalized to total phosphate (mean ± SD, from *n*=6 independent biological replicates). **E**) Disruption of cholesterol homeostasis in humans. Differentially expressed genes associated with cholesterol synthesis, uptake, efflux and trafficking in tumor biopsies and their respective matched normal tissue from human colorectal cancer patients (*n*=18). Fold change of gene expression was determined by comparison to normal tissue. Statistical significance determined by two-way ANOVA and post

Tukey's multiple comparison test. Different letters indicate significant differences between treatment groups (*P*<0.05). Source data are provided as a Source Data file.

Supplementary Figure 2

A)

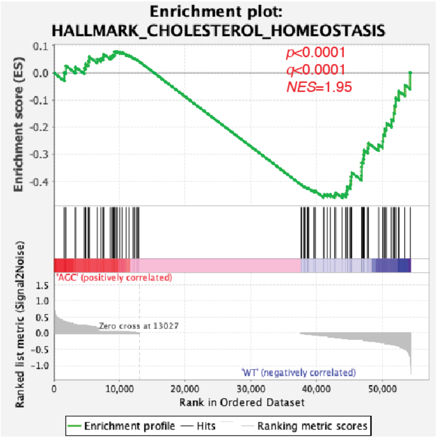

B)

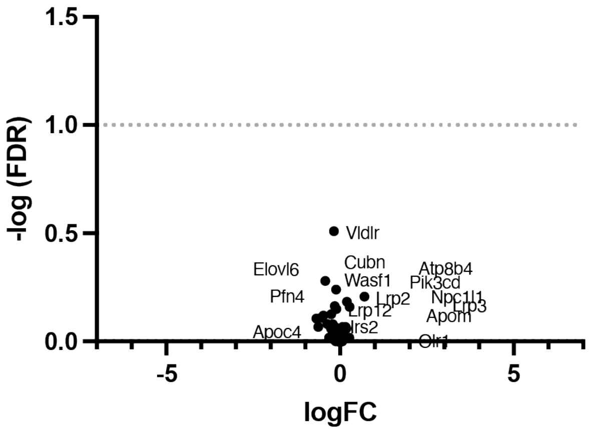

C)

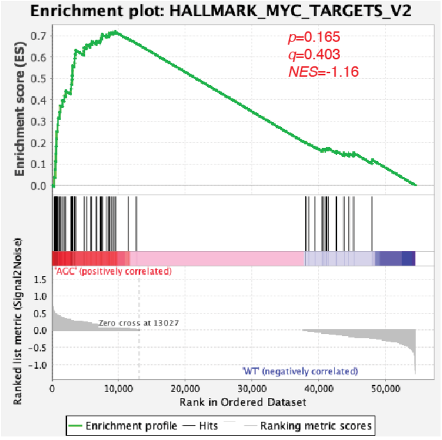

D)

GENE SET	SIZE	ES	NES	*p*-value	*q*-value
Hallmark Myc targets V2	57	0.72	1.95	<0.0001	<0.0001
Hallmark cholesterol homeostasis	75	-0.46	-1.16	0.165	0.403

Figure S2. Effect of one truncated APC allele on the expression of genes associated with cholesterol homeostasis and downstream Wnt signaling. A) Gene enrichment analysis of the cholesterol homeostasis pathway from AGC (Apc 580/+) het mice compared to GC (Apc +/+) mice. **B)** Volcano plot illustrating absence of differentially expressed genes in the cholesterol homeostasis pathway from AGC (Apc 580/+) het mice compared to GC (Apc +/+) mice (differential gene expression significance threshold=FDR>0.05) (Nom, nominal; FDR, false discovery rate; FC, fold change). **C)** Gene enrichment analysis of the downstream Wnt signaling pathway (βcat-related genes) from AGC (Apc 580/+) het mice compared to GC (Apc +/+) mice. **D)** Quantitative analysis from global gene set enrichment analysis in the cholesterol homeostasis and downstream Wnt signaling pathways from AGC (Apc 580/+) het mice compared to GC (Apc +/+) mice by normalized enrichment score. RNAseq analysis was performed using bulk RNA extracted from AGC (Apc 580/+) het mice (*n*=4 mice) and compared to GC (Apc +/+) mice (*n*=4 mice). ES, enrichment score; FDR, false discovery rate; NES, normalized enrichment score. Source data are provided as a Source Data file.

Supplementary Figure 3

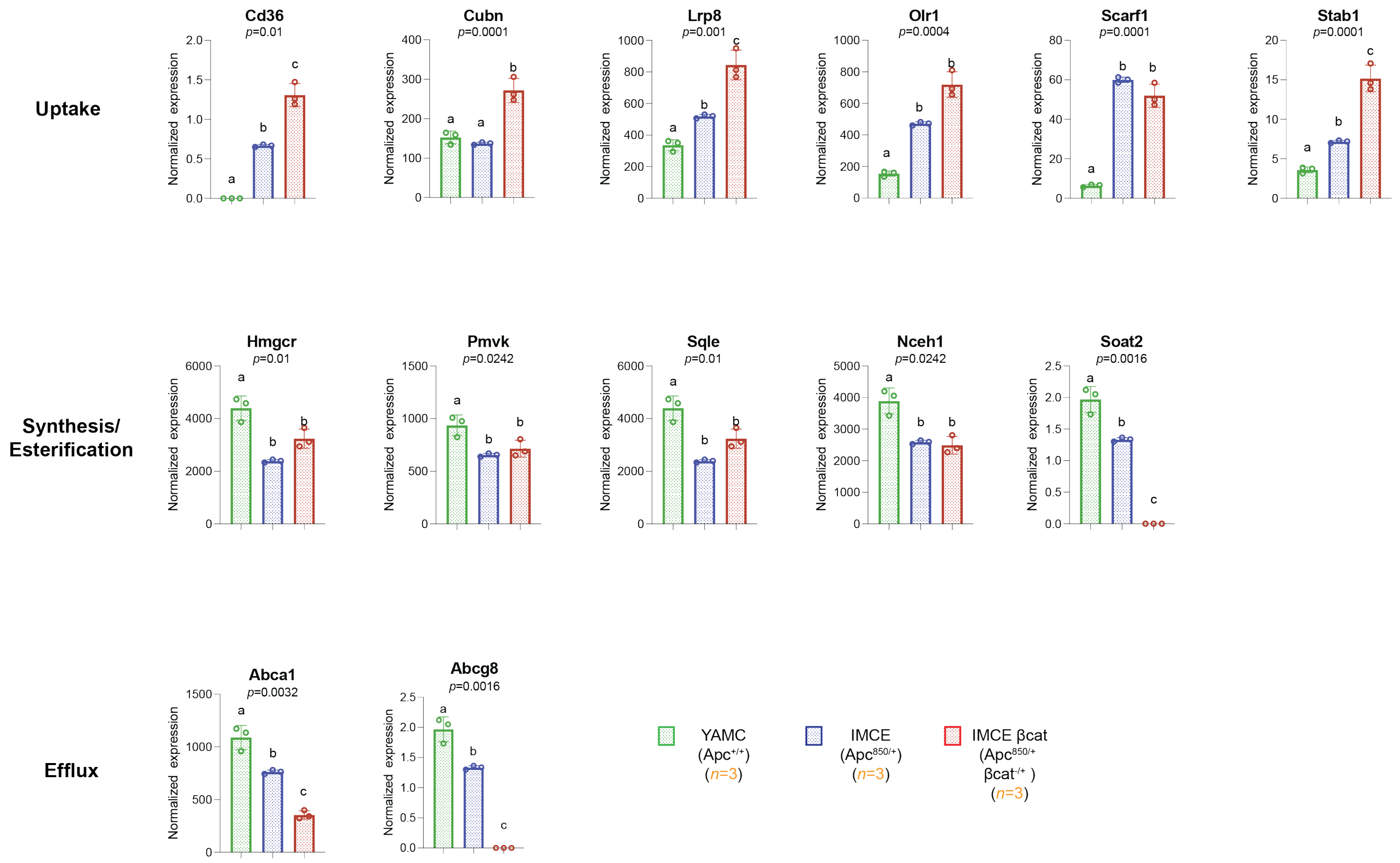

Figure S3. Oncogenic APC modulates the expression of cholesterol homeostasis-related genes in a dose-dependent manner. RNAseq analysis of bulk RNA from (Apc +/+), IMCE (Apc 850/+), IMCE βcat (Apc 850/+ βcat -/+) colonocytes. Statistical significance determined by two-way ANOVA and post Tukey's multiple comparison test. Error bars represent *n*=3 independent biological replicates (mean ± SD). Different letters indicate significant differences between treatment groups (*P*<0.05). Source data are provided as a Source Data file.

Supplementary Figure 4

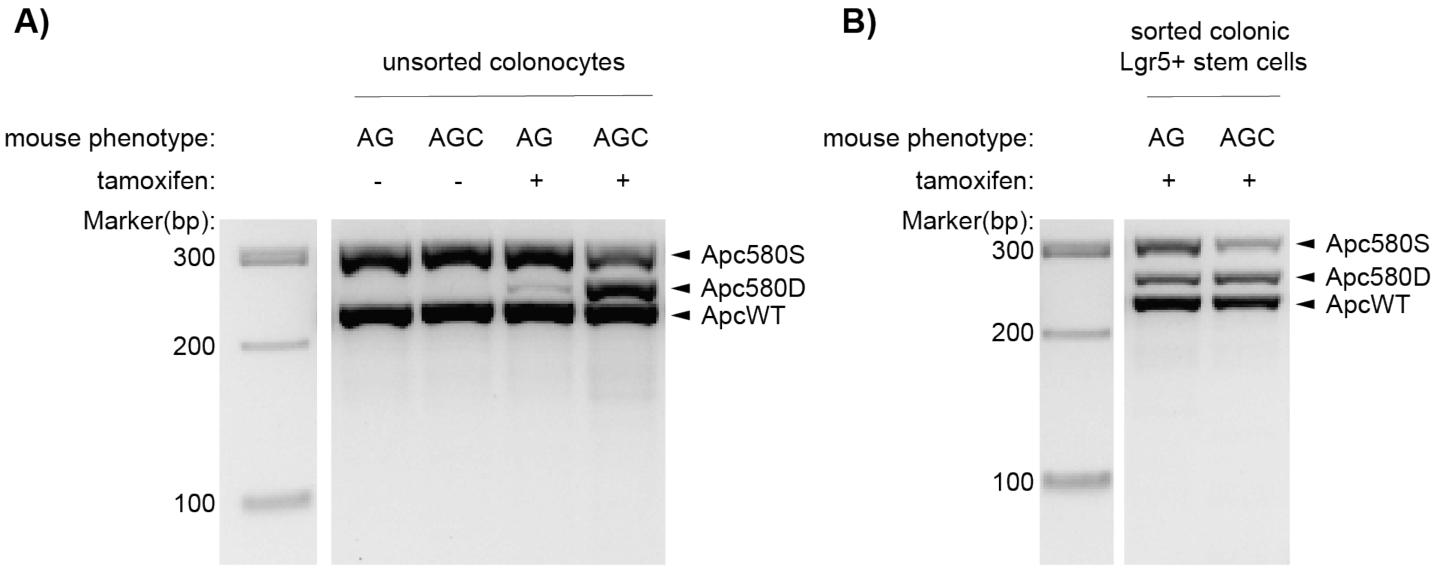

Figure S4. Analysis of Cre recombinase activation and loss of heterozygosity of *Apc* alleles in the mouse colon. Template DNA was extracted from **A**) whole population colonocytes or **B**) isolated Lgr5$^+$ colonic stem cells. Targeted DNA sequences were amplified by multiplex PCR. The WT Apc allele is represented by a 226-bp fragment. The floxed Apc allele (580S) is represented by 310-bp fragment, whereas the resulting inactive Apc allele (580D), targeted by Cre-recombinase, is represented by a 258-bp fragment. Oligonucleotide primer sequences have been provided as source data. Source data are provided as a Source Data file.

Supplementary Figure 5

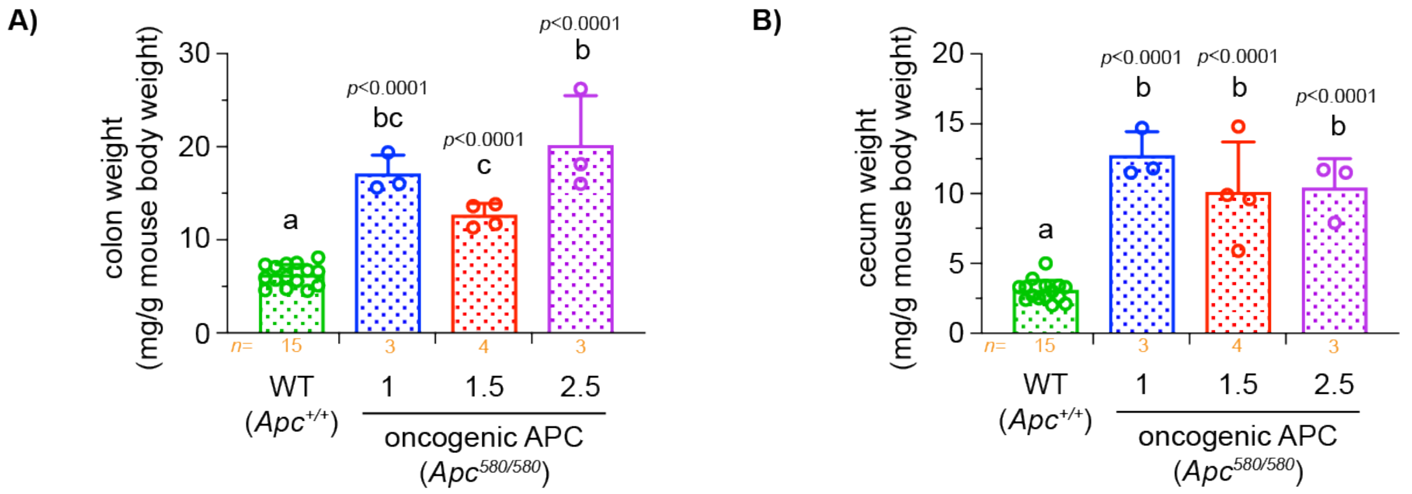

Figure S5. Effect of oncogenic truncated APC on mouse colon and cecum weight. Quantitative analysis of normalized **A)** whole colon and **B)** cecum weights from GC WT (Apc +/+) and AfGC (Apc 580/580) homo mice. Error bars represent data obtained from *n*=3-15 mice per group (mean ± SD). Statistical significance determined by one-way ANOVA and post Tukey's multiple comparison test. Different letters indicate significant differences between WT APC (control) and mutant APC (experimental) groups ($P<0.05$). Source data are provided as a Source Data file.

Supplementary Figure 6

A)

Group		Inflammation	Injury	Proliferation	Mucin
WT ($Apc^{+/+}$)		0,0,1	0,0,0	0,1,0	0,0,0
		0,1,0	0,1,0	0,1,0	0,0,0
		0,0,0	0,0,0	0,0,0	0,0,0
		1,1,1	0,1,1	0,0,1	0,0,0
oncogenic APC ($Apc^{580/580}$)	1	1,1,2	1,1,1	0,1,0	0,0,0
	1	2,2,2	1,1,1	1,1,1	2,2,1
	1	2,1,1	2,1,0	1,1,0	2,1,0
	3	3,2,2	2,1,1	2,1,1	2,1,1
	3	2,1,2	2,2,1	2,3,2	2,2,2
	3	3,2,2	2,1,2	3,2,2	2,2,1
	5	2,1,2	2,2,2	3,3,3	3,3,3
	5	2,2,2	2,2,2	2,3,3	2,3,3
	5	3,2,2	2,2,2	3,3,3	2,3,3

Scores:
central, mid, outer

B)

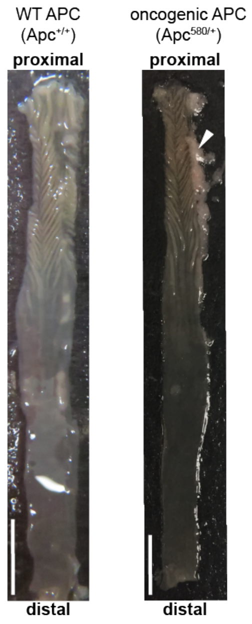

C)

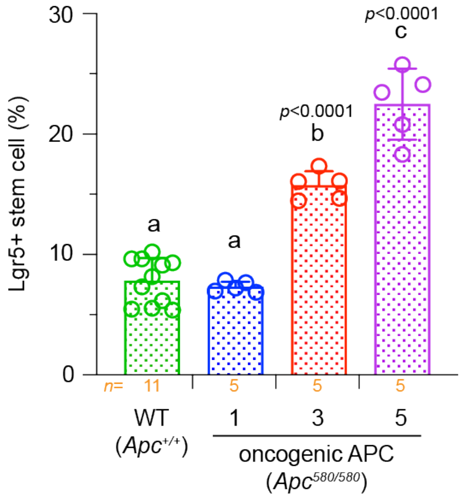

Figure S6. **Effect of oncogenic APC on colonic polyp formation and stem cell proliferation. A)** Swiss roll histology sections from AfGC (Apc 580/580) and GC (Apc+/+) mice were scored on a 0-3 scale (0 = normal to 3 = very abnormal). **B**) Representative AGC heterozygous mouse colonic polyp formation. Colon tissue from tamoxifen injected GC WT (Apc +/+, left) control and AGC het (Apc 580/+, right) mice exhibiting the effects of oncogenic APC, i.e., polyp formation. Arrowhead, mesenteric adipose tissue. Scale bars: 1 cm**. C)** Oncogenic APC alters colonic stem cell homeostasis. Flow cytometric quantitative analysis of colonic $Lgr5^+$ stem cells as a percentage of the colonocyte whole crypt population isolated from tamoxifen injected AfGC homo (Apc 580/580) mice. For all experiments, error bars represent stem cells obtained from n=5-11 mice (mean ± SD). Statistical significance determined by one-way ANOVA and post Tukey's multiple comparison test. Different letters indicate significant differences between treatment groups at each time point ($P<0.05$). Source data are provided as a Source Data file.

Supplementary Figure 7

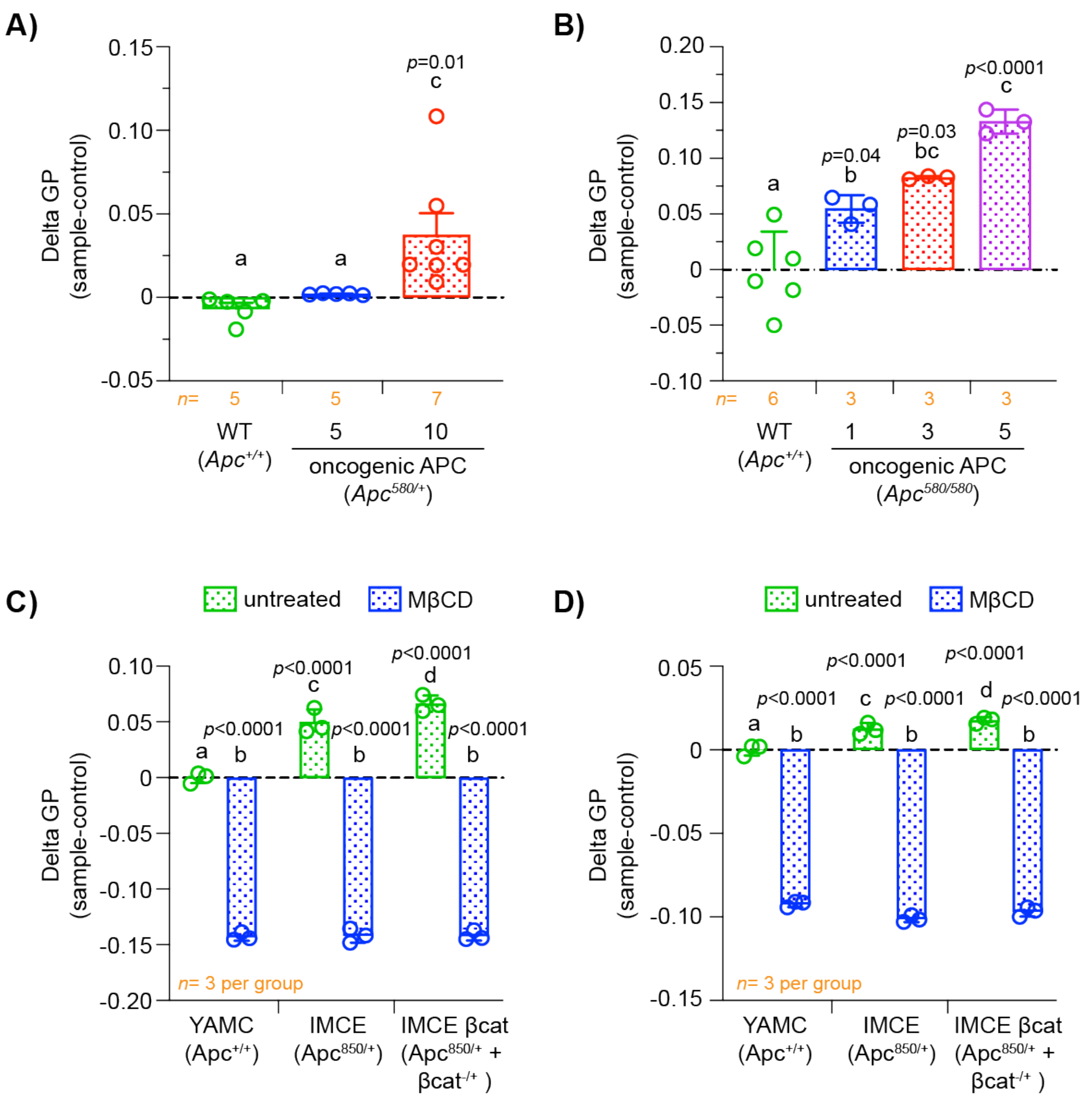

Figure S7. **Effect of oncogenic APC on plasma membrane rigidity.** Quantitative analysis of single colonocytes from tamoxifen injected **A)** AGC hetero (Apc 580/+) mice, **B)** AfGC homo (Apc 580/580) mice, **C)** cultured colonocytes (YAMC, IMCE and IMCE βcat) and their derived **D)** GPMVs. For all mouse experiments, error bars represent colonocytes obtained from *n*=3-7 mice (mean ± SD). Statistical significance determined by one-way ANOVA and post Tukey's multiple comparison test. For *in vitro* experiments, error bars represent cells and their derived GPMVs from *n*=3 independent biological replicates (mean ± SD). Statistical significance determined by two-way ANOVA and post Tukey's multiple comparison test. For all experiments, different letters indicate significant differences between treatment groups at each time point ($P<0.05$). Source data are provided as a Source Data file

Supplementary Figure 8

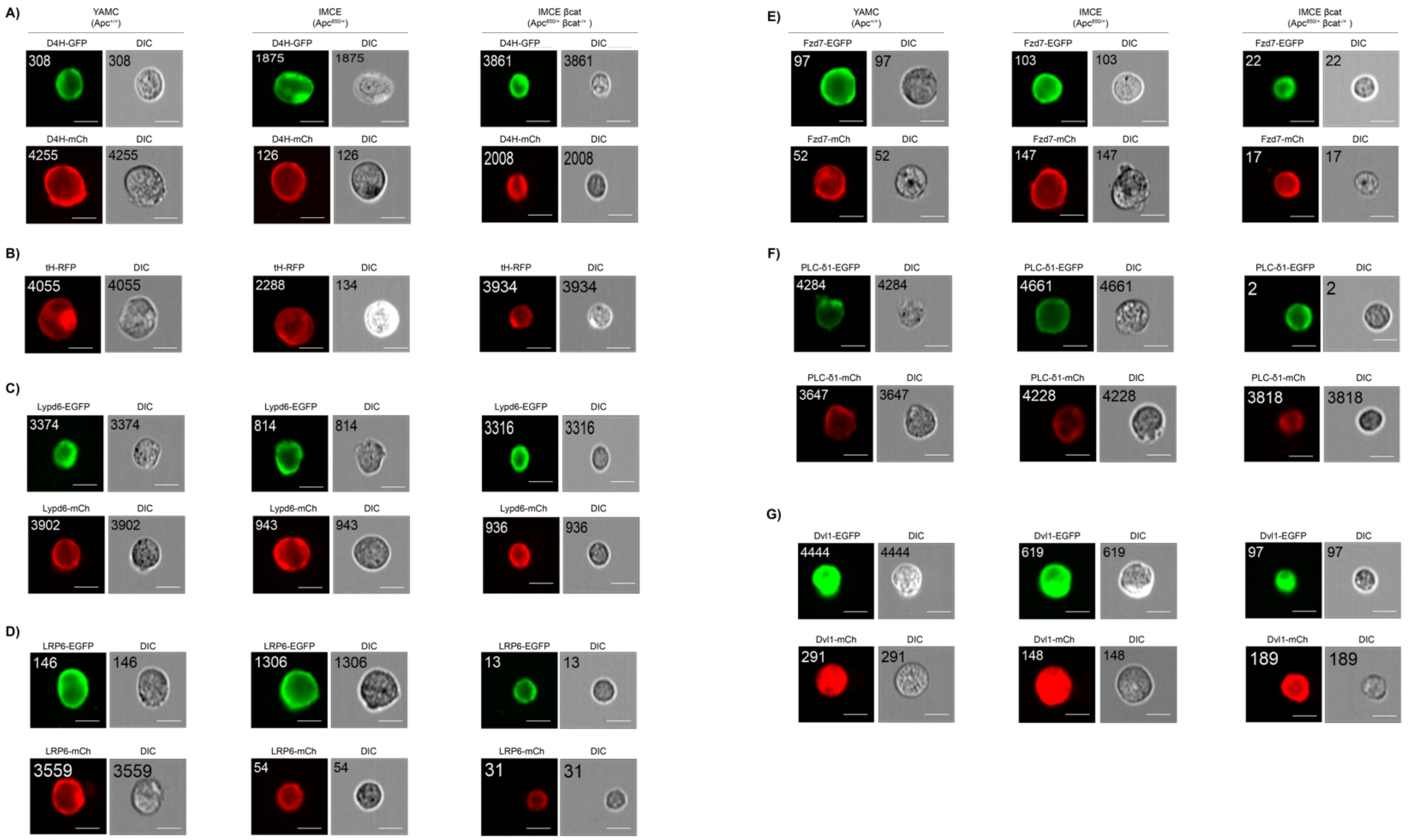

Figure S8. **Cellular expression of fluorescently-labeled recombinant Wnt receptors, effectors and markers in cultured colonocytes.** Representative flow cytometry fluorescence and DIC images of fixed YAMC ($Apc^{+/+}$, left), IMCE (Apc 850/+, middle) and IMCE βcat (Apc 850/+ βcat -/+, right) cells transfected with the **A)** cholesterol-sensing probe D4H, **B)** lipid raft marker tH-RFP, **C)** lipid raft resident Wnt potentiator Lypd6, Wnt receptors **D)** LRP6 and **E)** Fzd7, **F)** PI(4,5)P_2-sensing probe PLC-δ1, and **G)** Wnt effector Dvl1 fluorescently labeled with EGFP or mCherry. Values represent the number of single cells examined by flow cytometry analysis. Scale bars: 20 μm.

Supplementary Figure 9

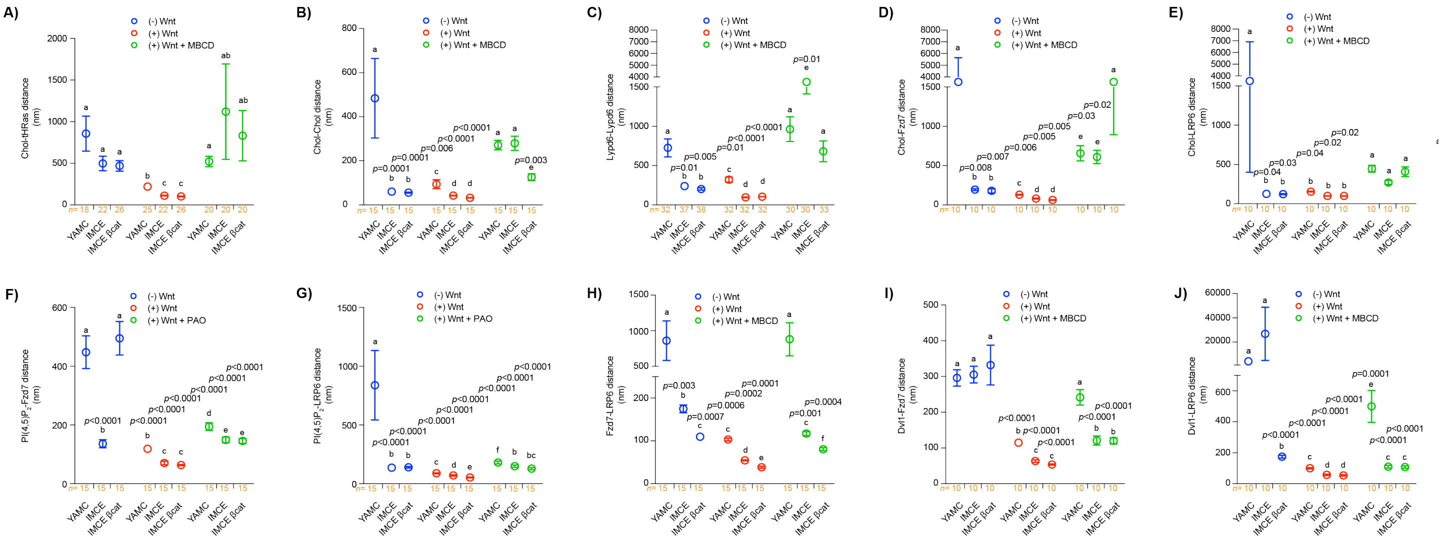

Figure S9. Oncogenic APC increased the proximity between members of the Wnt proteolipid condensate machinery. Quantitative analysis of the distance (r) between **A)** D4H-tHRas, **B)** D4H-D4H, **C)** Lypd6-Lypd6, **D)** D4H-Fzd7, **E)** D4H-LRP6, **F)** PLC-δ1-Fzd7, **G)** PLC-δ1-LRP6, **H)** Fzd7-LRP6, **I)** Dvl1-Fzd7 and **J)** Dvl1-LRP6 FLIM-FRET probes. To measure the distance (r) between FLIM-FRET probes, we used our fluorescence lifetime results and the lifetime dono-aceptor (τ_{DA}) equation as described in the materials and methods section. Conditions are the same as their corresponding FLIM-FRET experiment. the apparent FRET distance (r) was calculated from FLIM data averaged per field of view (mean ± SD, *n*=10-38 field of views per condition). Statistical significance determined by two-way ANOVA and post Tukey's multiple comparison test. For all experiments, different letters indicate significant differences between treatment groups at each time point (*P*<0.05). Source data are provided as a Source Data file.

Supplementary Figure 10

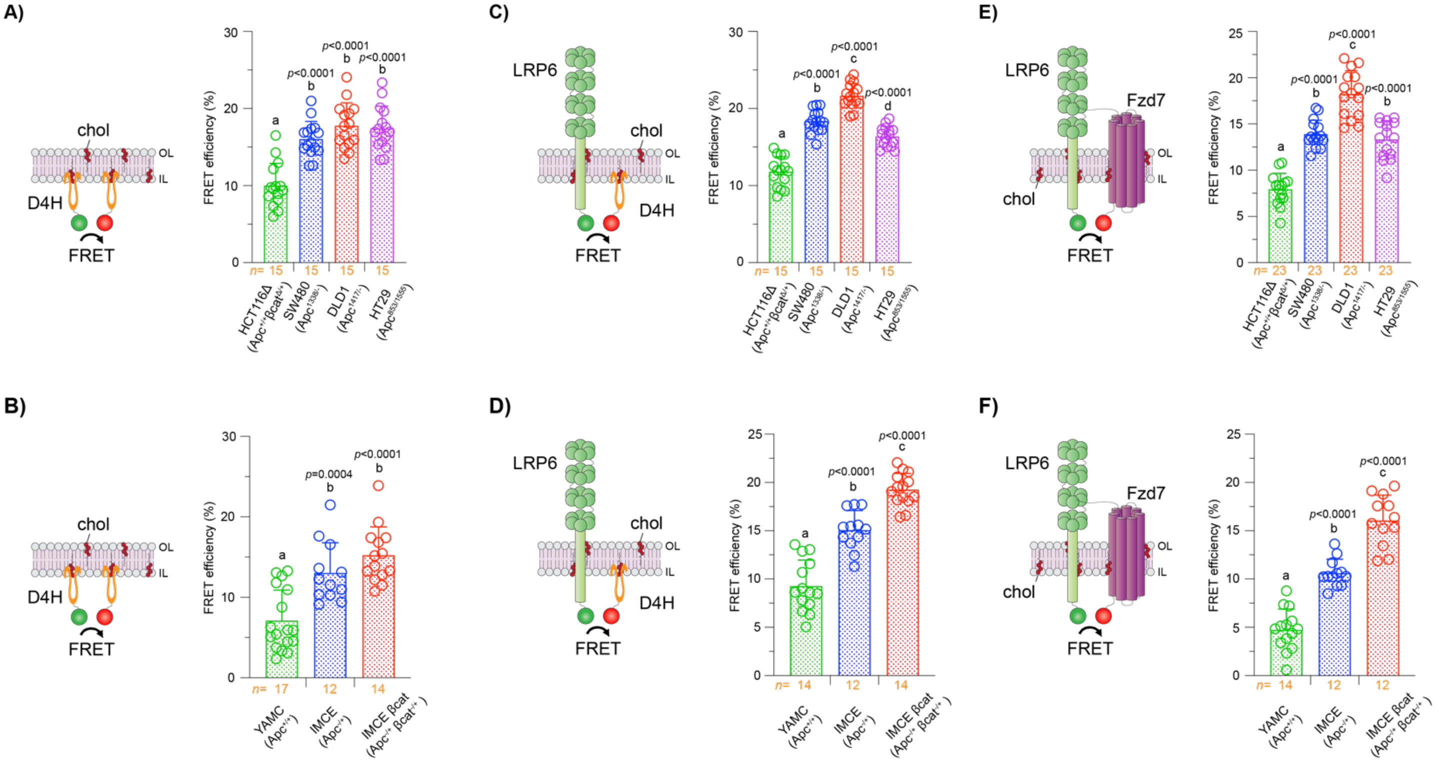

Figure S10. Effect of oncogenic truncated APC on plasma membrane lipid-lipid, protein-lipid and protein-protein interactions. To assess the effects of oncogenic APC on lipid raft resident molecules, cells were transfected, fixed with 4% PFA and 0.2% glutaraldehyde and imaged. Colonocytes co-expressing EGFP- and mCherry-tagged **A** and **B)** D4H, **C** and **D)** LRP6 and D4H and **E** and **F**) LRP6 and Fzd7 were used to for FLIM-FRET analyses. For FLIM-FRET experiments, the apparent FRET efficiency was calculated from FLIM data averaged per field of view (mean ± SD, *n*=12-23 field of views containing 3-8 cells were examined per condition). Statistical significance was determined by one-way ANOVA and post Tukey's multiple comparison test. Different letters indicate significant differences between treatment groups (*P*<0.05).

Supplementary Figure 11

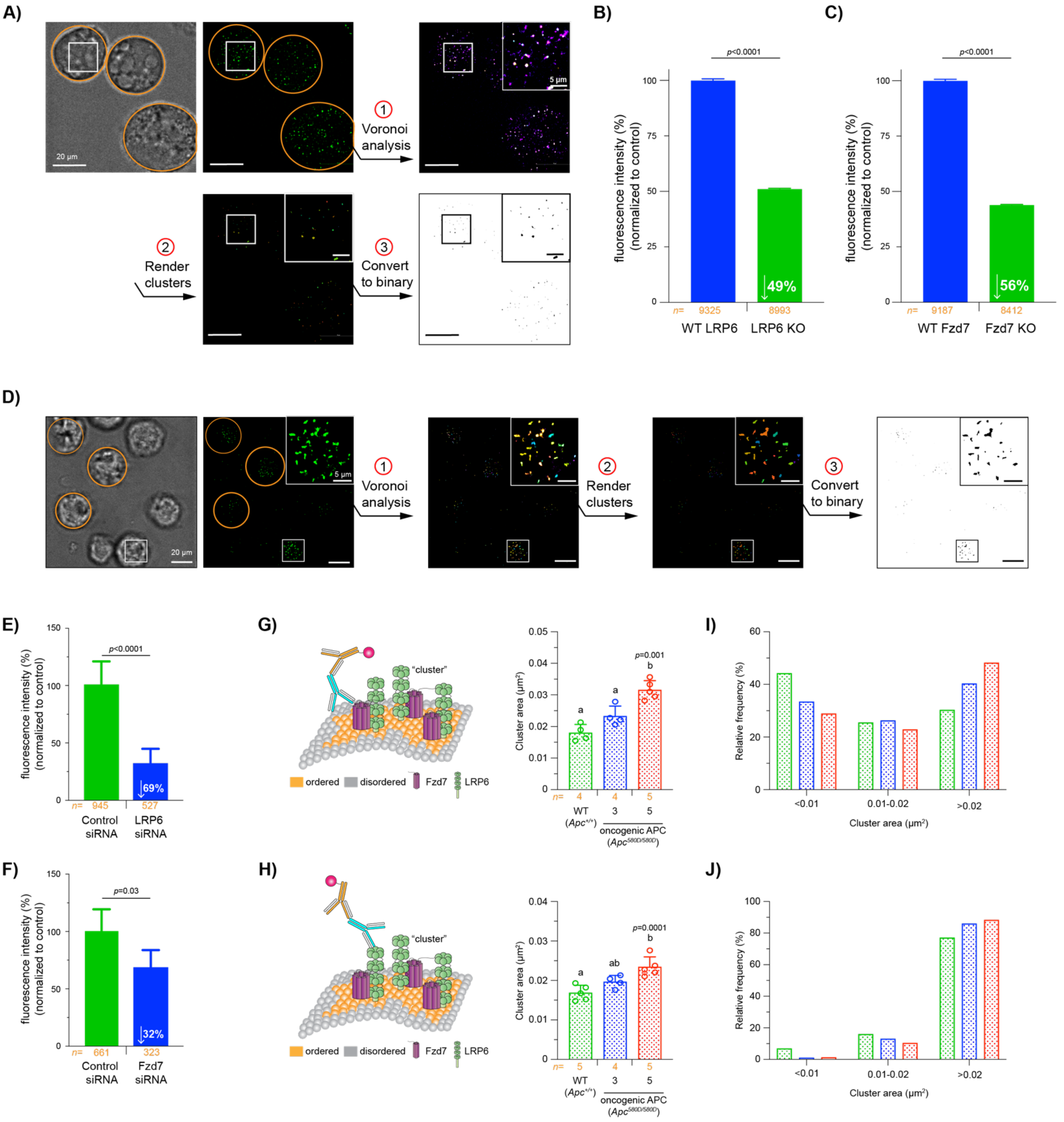

Figure S11. ***In vivo*** **super-resolution analysis of Wnt condensates using STORM.** Representative images of fixed isolated single colonocytes from **A)** CRC-PDOs and **D)** tamoxifen injected AfGC homo (Apc 580/580) mice labeled with mouse primary monoclonal LRP6 Alexa Fluor 647 or rat LRP6 antibody and secondary donkey anti-rat IgG tagged with CF568 for STORM imaging, respectively. Numbers represent the sequential steps of super resolution analysis to generate quantifiable single cluster regions from a STORM fluorescence image. Orange circle, single colonocyte outline; white square, zoomed area shown in inset. Scale bars: 20 µm; 5 µm (inset). Characterization of LRP6 and Fzd7 STORM antibody specificity. **B** and **C)** HAP1 LRP6-knock out (KO) and Fzd7-KO cells incubated with mouse primary monoclonal LRP6 Alexa Fluor 647 and rat primary monoclonal Fzd7 Alexa Fluor 647 as well as **E** and **F)** siRNA knockdown of LRP6 and Fzd7 levels in cultured colonocytes (YAMC) labeled with primary monoclonal rat Fzd7 or LRP6 antibody and secondary donkey anti-rat IgG tagged

with CF568. The fluorescence intensity of each antibody was quantified using flow cytometry. Error bars represent fluorescence intensity of all cells **B** and **C)** *n*=8412-9325 and **E** and **F**) *n*=323-945 in each group, normalized to control (mean ± SD). Statistical significance determined by two-tailed unpaired t-test ($P<0.05$). Quantitative analysis of **G**) Fzd7 and **H**) LRP6 cluster area and **I)** Fzd7 and **J)** LRP6 cluster size relative frequency in isolated single colonocytes from GC (Apc +/+) and AfGC (Apc 580/580) mice. Cluster area was calculated from STORM data averaged per mouse and the respective relative frequency was calculated from individual cluster distribution data (mean ± SD, from *n*=4-5 mice; number of individual clusters examined per treatment group are the following Fzd7: WT=2,472, AfGC 3 wk=1,854, AfGC 5 wk=3,716; LRP6: WT=2,100, AfGC 3 wk=2,330, AfGC 5 wk=4,115 from four wells per group. Different letters indicate significant differences between treatment groups ($P<0.05$). Source data are provided as a Source Data file.

Supplementary Figure 12

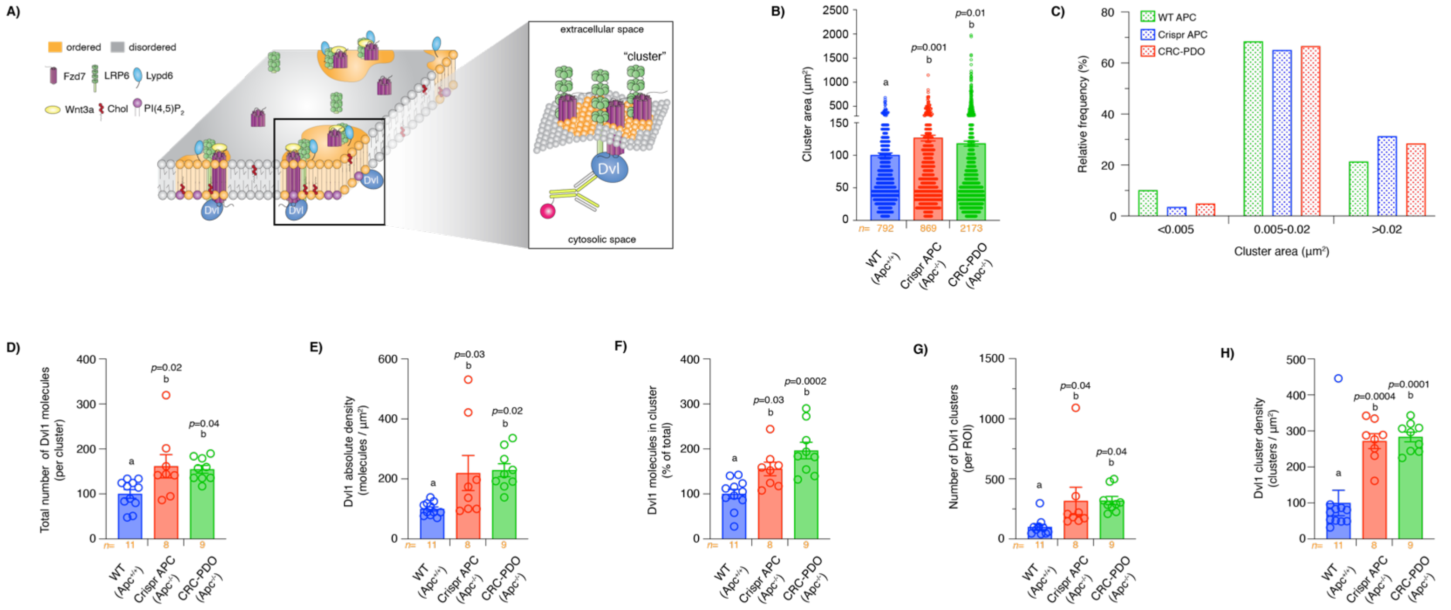

Figure S12. Oncogenic APC modulates a cytosolic protein (Dvl) that forms part of the Wnt proteolipid condensate machinery. For *in vivo* STORM imaging experiments, isolated single colonocytes from PDOs were fixed and labeled with primary monoclonal mouse Dvl1 antibody fluorescently labeled with Alexa Fluor 647. **A)** Model of the formation of Wnt proteolipid condensates in ordered plasma membrane nanodomains examined via STORM imaging. Quantitative analysis of Dvl1 **B)** cluster area, **C)** cluster area relative frequency, **D)** total number of Dvl1 molecules inside clusters, **E)** Dvl1 molecule absolute density, **F)** percentage of Dvl1 molecules forming part of clustered regions, **G)** total number of Dvl1 clusters, and **H)** cellular Dvl1 cluster density in isolated single colonocytes from PDOs, respectively. Cluster area was calculated from STORM data averaged per region of interest (ROI) (mean ± SD, from *n*=792-2173 ROIs) and the respective relative frequency was calculated from individual cluster distribution data. Data associated with the number of single receptor molecules, receptor clusters and their density were calculated from raw fluorescence intensity images converted to text (.txt) *x-y* coordinate files using Clus-Doc (mean ± SD, from *n*=8-11 field of views). Different letters indicate significant differences between treatment groups ($P<0.05$). Source data are provided as a Source Data file.

Supplementary Figure 13

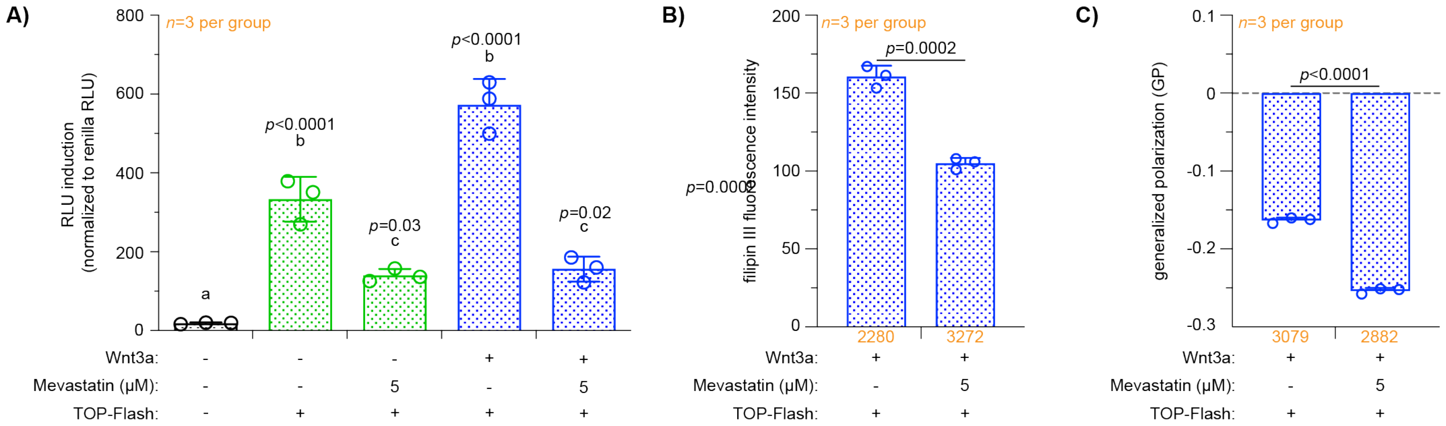

Figure S13. **Use of mevastatin to suppress downstream Wnt activation.** IMCE (Apc 850/+) cells co-transfected with the βcat-TCF firefly luciferase reporter, TOP-Flash, and Renilla luciferase reporter (transfection efficiency control) were treated with mevastatin (5 µM, 24 h), stimulated with Wnt3a-conditioned media for 24 h and used to measure βcat activation, cholesterol levels and plasma membrane rigidity. **A)** Quantitative analysis of βcat activation. βcat-mediated firefly luciferase induction was measured using a Dual-Luciferase® Reporter Assay system. βcat-mediated firefly luciferase induction was calculated from luminescence data and normalized to transfection efficiency using Renilla luciferase. Luminescence data from firefly luciferase (mean ± SEM, from *n*=3 independent biological replicates, ~3x10^5 cells were examined per condition). Statistically significant differences between conditions were determined using one-way ANOVA and post Tukey's multiple comparison test. Different letters indicate significant differences between treatment groups (*P*<0.05). **B)** Quantitative analysis of cholesterol levels by flow cytometry. Fixed IMCE colonocytes were incubated with filipin III (50 µg/mL) for 45 min in the dark, washed and imaged. Error bars represent cells from *n*=3 independent biological replicates (mean ± SEM, from *n*=3 independent biological replicates, total cells analyzed provided below bars). Statistical significance was determined by two-tailed unpaired t-test (P<0.05). **C)** Quantitative analysis of plasma membrane rigidity by flow cytometry. IMCE colonocytes were incubated with Di-4 (1 µM) and rapidly imaged. Error bars represent cells from *n*=3 independent biological replicates (mean ± SEM, from *n*=3 independent experiments, total cells analyzed provided below bars). Statistical significance determined by two-tailed unpaired t-test (*P*<0.05). Source data are provided as a Source Data file.

Supplementary Figure 14

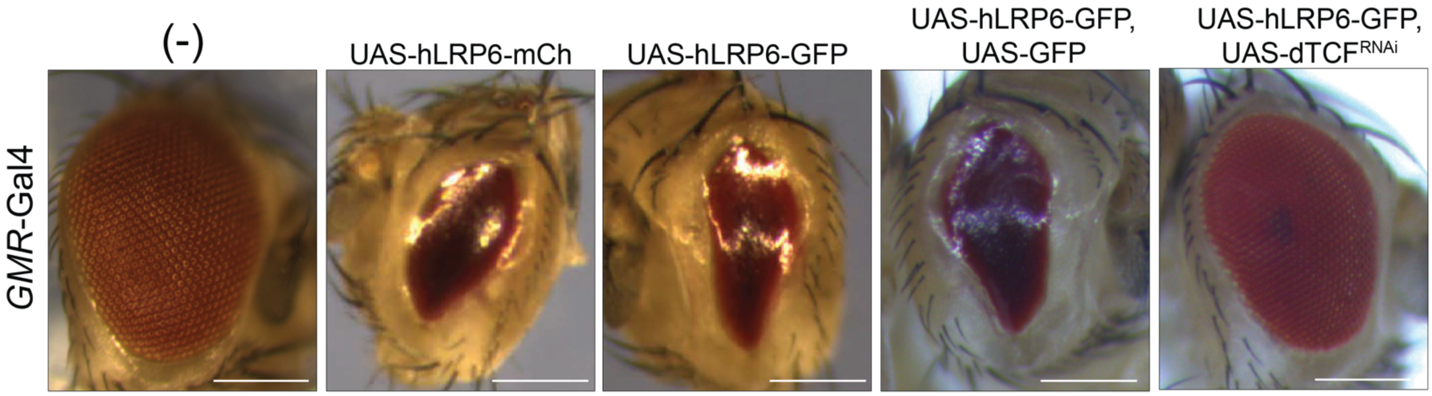

Figure S14. **Effect of hLRP6 over-expression on Wnt signaling activity in Drosophila**. Light micrographs of Drosophila adult eyes are shown. Expression of human LRP6 (GMR-Gal4 > UAS-hLPR6-mCh or GMR-Gal4 > UAS-hLPR6-GFP) in developing eyes under the control of GMR promoter induces a ‘glassy eye’ phenotype compared to the control flies (GMR-Gal4>(-); Ctrl.). This phenotype is suppressed by attenuating the transcription factor dTCF using RNAi (GMR-Gal4>UAS-hLPR6-GFP, UAS-dTCF^{RNAi}) compared to control flies (GMR-Gal4>UAS-hLPR6-GFP, UAS-GFP). Scale bars: 500 µm.

Supplementary Figure 15

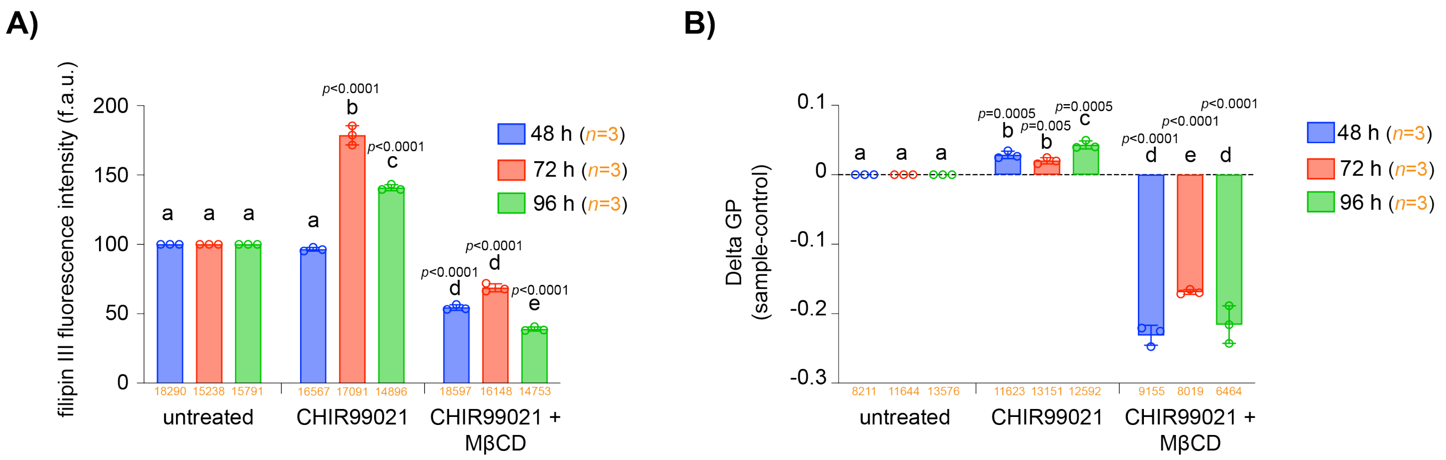

Figure S15. Effect of a GSK3β inhibitor on the levels of plasma membrane free cholesterol and rigidity. Quantitative analysis of plasma membrane **A)** free cholesterol and **B)** rigidity. YAMC (Apc+/+) colonocytes were incubated with 2.5 μM CHIR99021 for the indicated times. Following CHIR99021 incubation, cells were treated 5 mM MβCD as indicated, washed, immediately labeled with **A)** 1 μM Di4 and imaged via flow cytometry or **B)** fixed, washed, labeled with 50 µg/mL filipin III for 45 min, washed and imaged via flow cytometry. Error bars represent fluorescence intensity of all cells (n=3 independent biological replicates, total number of cells analyzed provided below bars) per condition, normalized to untreated (mean ± SD). Statistical analysis was performed using a two-way ANOVA and post Tukey's multiple comparison test. Different letters indicate significant differences between treatment groups ($P<0.05$). Source data are provided as a Source Data file.

Supplementary Figure 16

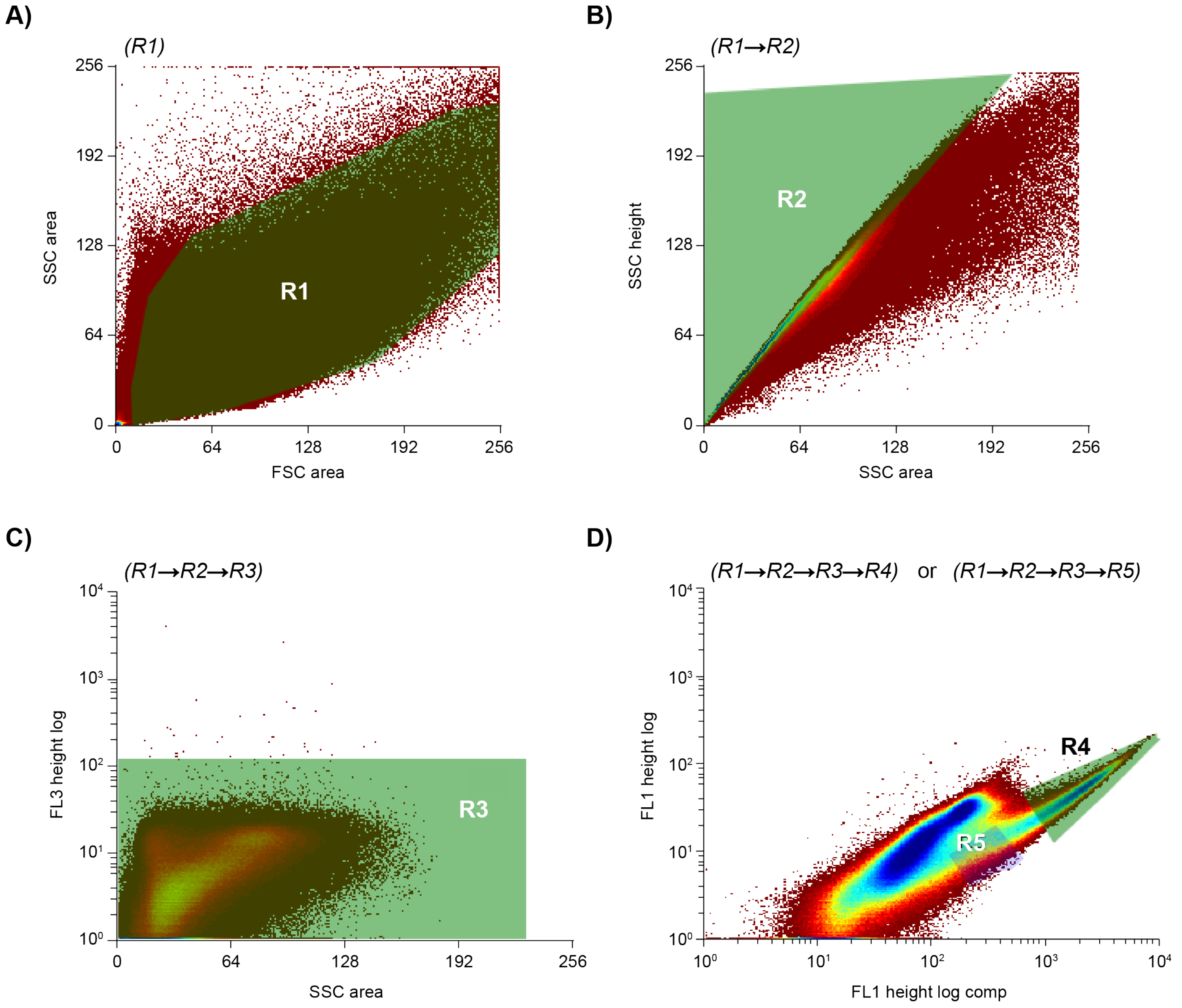

Figure S16. Isolation of Lgr5$^+$ colonic stem cells (CSC) via fluorescence-activated cell sorting (FACS). A specific in-house protocol was generated to isolate Lgr5$^+$ CSCs expressing GFP (GFP$^+$), under regulation of a Lgr5 promoter, from a population of single primary colonocytes obtained from mouse crypts. CSCs isolated with this protocol were utilized to perform *in vivo* CSC-specific quantitative analysis of plasma membrane free cholesterol and rigidity, and CSC proliferation (**Figures 4H** and **5D**, and **Figure S6C**, respectively). **A**) Representative flow cytometric dot plots of the first gating step (R1) applied to single primary colonocyte samples, which allows the removal of small and large debris as well as very large cell aggregates leading to the isolation of a homogenous single cell (hSC) population. **B**) Representative flow cytometric dot plots of the second gating step (R2) applied to the hSCs population, which allows the removal of doublets and small cell aggregates leading to the isolation of a highly hSC (hhSC) population. **C**) Representative flow cytometric dot plots of the third gating step (R3) applied to the hhSC population, which allows the removal dead cells leading to the isolation of live hhSCs (lhhSC). **D**) Representative flow cytometric dot plots of the third gating step applied on lhhSCs, which selects lhhSCs expressing high (R4) or low (R5) fluorescence levels of GFP. In this case, Lgr5$^+$ CSCs and CSC daughters correspond to the R4 and R5 regions, respectively. For our experiments, we employed only the population isolated from using sorting logic R4 (R1→R2→R3→R4). SSC=side scatter light; FSC=forward scatter light, FL1=green fluorescence channel detector (525/30 filter); FL3= red fluorescence channel detector (615/25 filter).

Supplementary Table 1

**De novo cholesterol synthesis**
ACAA1A
ACAA1B
ACAA2
CYP51A
DHCR24
DHCR7
EBP
FDFT1
FDPS
GGPS1
HMGCR
HMGCS1
HMGCS2
HSDT7B7
IDI1
MVD
MVK
NSDHL
PMVK
SC4MDL
SC5DL
SQLE
TM7SF2
**Sphingolipid de novo synthesis**
CERS1
CERS2
CERS3
CERS4
CERS5
CERS6
DEGS1
DEGS2
KDSR
SGPL1
SPHK1
SPHK2
SPTA1
SPTB

**Phospholipid ethanolamine pathway**
CDS
CDS2
CHKA
CHKB
CTPS
CTPS2
ECT
ETNK1
ETNK2
SELENOI
**Phospholipid choline pathway**
PEMT
PTDSS1
PTDSS2
**Structural proteins**
APOA1
APOA2
APOA4
APOA5
APOB
APOC1
APOC3
APOC4
APOD
APOE
APOF
APOH
APOM
CAV1
CAV2
CAV3
CAVIN1
CAVIN2
CAVIN3
CAVIN4
**Cholesterol transporters**
ABCA1

ABCA7
ABCB4
ABCG1
ABCG4
ABCG5
ABCG8
LDLR
LRP1
LRP10
LRP11
LRP12
LRP2
LRP3
LRP4
LRP5
LRP6
LRP7
LRP8
**Phospholipid transporters**
ATP10A
ATP10B
ATP10D
ATP11A
ATP11B
ATP11C
ATP11C
ATP8A1
ATP8A2
ATP8B1
ATP8B2
ATP8B4
ATP8B5
ATP9A
ATP9B
PLSCR1
PLSCR2
PLSCR3
PLSCR4
TMEM30A
**Sphingolipid transporters**

ABCA2
CPTP
PLEKHA8
STARD11
**Lipases/Transporter proteins**
LIPE
LIPG
LPL
PLTP
**Fatty acid de novo synthesis**
ACACA
ACACB
MCAT
**Fatty acid elongases**
ELOVL1
ELOVL2
ELOVL3
ELOVL4
ELOVL5
ELOVL6
ELOVL7
**Fatty acid desaturases**
FADS1
FADS2
FADS3
FADS6
HACD1
HACD2
HACD3
HACD4
SCD1
SCD2
SCD3
SCD4
**Lipoprotein receptors**
APOER
CD36
CDH13
COLEC12
CUBN

CXCL16
LDLR
LDLRAP1
LRPAP1
NPC1L1
NUMB
ORL1
P2RY13
SCARB1
SCARF1
SCARF2
SORL1
SORT1
STAB1
STAB2
VLDLR
**Intracellular cholesterol trafficking**
GRAMD1A
GRAMD1B
GRAMD1C
INSIG1
INSIG2
MBTPS1
MBTPS2
NCEH1
OSBP1
OSBPL1A
OSBPL5
PEX2
PEX5
SCAP
SMPD1
SNX17
STAR
STARD4
STARD7
STARD3
STARD6
NPC1
NPC2
SORL1
**Unfolded protein response**

EIF2AK3
ERN1
ERN2
HERPUD1
HERPUD2
XBP1
ATF6
HSPA5
MAP3K5
ATF4
**Autophagy**
ATG5
ATG12
ATG13
ATG16
ATG101
MALP1LC3B
PIK3R4
RB1CC1
ULK1
UVRAG
**Insulin**
IRS1
IRS2
IRS3
PIK3R1
PIK3R2
PIK3CA
PIK3CD
AKT1
AKT2
AKT3
MTOR
FOXO1
GSK3B
CAP1
SRC
**Transcription factors**
CNBP
NRIH3
PPARGC1A
PRMD16
RXRA
RXRB

RXRG
SREBF1
SREBF2
TRERF1
PPARA
PPARD
PPARG
NRIH4
**Lipoprotein related proteins**
HDLBP
PCSK9
**IPA predicted networks**
XDH
APP
CXCL16
UCP1
UCP2
UCP3
**Lipid receptors**
FFAR4
**Adipokine receptors**
ADIPOR1
ADIPOR2

**Cytoskeleton**
ACTB
ACTA1
ACTA2
PFN1
PFN2
PFN4
ACTR2
ACTR3
WAS
FMN1
FMN2
PLCG1
PLCG2
PLCB1
PLCB2
PLCB3
PLCB4
PLCD1
PLCD3
PLCD4
WASL
RHO
RHOA
FCHSD1

FCHSD2
WASF1
WASF2
WASF3
MTM1
TUBB
TUB1A
TUBG1
TUBB3
TUBA3C
TUBA3E
TUBA3C
TUBB1
**Cholesterol esterification**
TUBB1
ACAT1
ACAT2
SOAT1
SOAT2
**Oxysterol synthesis**
CYP7B1
CYP11A1
CYP27A1
CYP39A1

Table S1. Key genes involved in plasma membrane biophysical homeostasis. Gene target list generated from cholesterol, sphingolipid and fatty acid metabolic literature (~ 290 genes) to predict key upstream regulators.

Supplementary Table 2

A)

IMCE (Apc-/+) / YAMC (Apc+/+)

Autophagy			
	P Value	FDR	FC
Rb1cc1	3.86E-05	1.97E-03	0.39
Beta oxidation			
	P Value	FDR	FC
Acaa1b	9.94E-07	8.36E-05	0.14
Acaa2	1.56E-04	6.16E-03	1.89
Cholesterol de novo synthesis			
	P Value	FDR	FC
Ebp	1.40E-03	3.63E-02	1.73
Cholesterol intracellular trafficking			
	P Value	FDR	FC
Pcsk9	6.74E-10	1.08E-07	0.14
Soat2	1.19E-05	7.37E-04	10.75
Mboat2	1.36E-05	8.21E-04	0.30
Tspo	1.82E-05	1.07E-03	2.04
Sorl1	2.20E-05	1.24E-03	12.75
Stard6	4.00E-04	1.34E-02	3.32
Ces2e	5.31E-04	1.69E-02	7.25
Cholesterol uptake			
	P Value	FDR	FC
Colec12	7.25E-11	1.29E-08	0.19
Scarf1	8.03E-08	8.73E-06	10.51
Sort1	4.97E-06	3.47E-04	0.38
Olr1	4.29E-06	0.000306	3.47
Vldlr	9.88E-05	4.28E-03	0.38
Stab2	1.56E-03	3.93E-02	24.84
Cytoskeleton			
	P Value	FDR	FC
Wasf3	5.11E-22	3.17E-19	0.01
Acta1	1.89E-03	4.55E-02	10.62
Glycolysis			
	P Value	FDR	FC
Eno1b	2.18E-66	8.29E-63	0.005
Ldhb	1.42E-09	2.16E-07	0.14

GTPases			
	P Value	FDR	FC
Stard13	3.91E-04	1.32E-02	0.48
Ketogenesis			
	P Value	FDR	FC
Hmgcll1	1.36E-07	1.36E-05	55.07
Lipases			
	P Value	FDR	FC
Lpl	1.41E-22	9.36E-20	0.08
Lysophosphatidic acid metabolism			
	P Value	FDR	FC
Lpar1	1.84E-05	1.08E-03	0.42
Oxidation of sterols and retinoids			
	P Value	FDR	FC
Dhrs3	1.74E-05	1.03E-03	0.27
Cyp26b1	3.05E-04	1.08E-02	3.87
Cyp27a1	4.24E-04	1.41E-02	6.85
Rpe65	1.26E-03	3.35E-02	42.29
Phospholipases			
	P Value	FDR	FC
Pla1a	4.55E-04	1.48E-02	3.94
Plcb1	3.88E-03	7.71E-02	2.76
Atp10d	2.26E-25	1.77E-22	0.09
Structural lipoprotein			
	P Value	FDR	FC
Apol11a	4.31E-21	2.39E-18	0.002
Apol10a	1.41E-09	2.16E-07	0.04
Transcription factor			
	P Value	FDR	FC
Rxra	1.59E-07	1.56E-05	2.44
Pparg	1.57E-03	3.96E-02	3.70
Cebpa	2.37E-03	5.40E-02	3.77
Unfolded protein response			
	P Value	FDR	FC
Map3k5	1.99E-08	2.46E-06	13.41

B)

IMCE βcat (Apc-/+ βcat-/+) / YAMC (Apc+/+)

Beta oxidation			
	P Value	FDR	FC
Acaa1b	1.43E-07	1.18E-05	0.12
Acaa2	5.26E-04	1.15E-02	1.79
cAMP metabolism			
	P Value	FDR	FC
Cd38	3.66E-05	1.33E-03	27.63
Cap1	1.59E-03	2.70E-02	0.48
Cholesterol de novo synthesis			
	P Value	FDR	FC
Ebp	2.59E-03	3.94E-02	1.68
Cholesterol efflux			
	P Value	FDR	FC
Abca1	5.54E-03	7.04E-02	0.31
Abcg8	6.02E-03	7.50E-02	0.06
Cholesterol intracellular trafficking			
	P Value	FDR	FC
Pcsk9	3.07E-10	4.31E-08	0.14
Sorl1	5.38E-08	5.01E-06	26.51
Soat2	2.14E-07	1.69E-05	17.51
Mboat2	1.50E-05	6.48E-04	0.30
Abca2	1.28E-03	2.28E-02	2.20
Tspo	1.28E-03	2.28E-02	1.71
Cholesterol uptake			
	P Value	FDR	FC
Colec12	1.40E-07	1.15E-05	0.28
Sort1	9.97E-07	6.49E-05	0.33
Scarf1	3.23E-06	1.79E-04	7.58
Lrp8	7.02E-04	1.44E-02	2.38
Lrp2	1.00E-03	1.89E-02	0.45
Olr1	5.72E-08	5.28E-06	4.41
Cytoskeleton			
	P Value	FDR	FC
Wasf3	5.41E-23	3.66E-20	0.001
Tubb3	6.41E-08	5.83E-06	8.77
Acta2	8.31E-04	1.63E-02	0.12

Desaturases			
	P Value	FDR	FC
Scd3	1.01E-04	2.99E-03	8.08
Fatty acid metabolism			
	P Value	FDR	FC
Hacd4	1.37E-06	8.47E-05	0.18
Glycolysis			
	P Value	FDR	FC
Eno1b	6.73E-60	2.05E-56	0.009
Ldhb	2.66E-10	3.82E-08	0.13
GTPases			
	P Value	FDR	FC
Stard13	8.34E-04	1.64E-02	0.50
Ketogenesis			
	P Value	FDR	FC
Hmgcll1	1.21E-04	3.50E-03	21.59
Lipases			
	P Value	FDR	FC
Lpl	7.97E-19	3.68E-16	0.10
Lysophosphatidic acid metabolism			
	P Value	FDR	FC
Lpar1	4.86E-06	2.57E-04	0.40
Lpar4	4.33E-05	1.52E-03	0.21
Lpcat2	8.83E-03	9.85E-02	0.38
Mitochondrial proteins			
	P Value	FDR	FC
Ucp2	6.52E-03	7.96E-02	0.25
Nitric oxide synthesis			
	P Value	FDR	FC
Arg1	2.49E-05	0.000968	29.97
Oxidation of sterols and retinoids			
	P Value	FDR	FC
Dhrs3	4.78E-05	1.65E-03	0.30
Cyp26b1	1.50E-04	4.17E-03	4.09
Cyp27a1	7.45E-03	8.76E-02	4.58

Phospholipases			
	P Value	FDR	FC
Pld3	1.17E-04	3.40E-03	0.41
Pla1a	8.15E-04	1.61E-02	3.68
Plcb4	4.00E-03	5.51E-02	0.17
Phospholipid metabolism			
	P Value	FDR	FC
Pfn4	2.96E-05	1.12E-03	0.07
Pcyt1b	5.23E-03	6.74E-02	3.66
Atp10d	1.46E-27	1.43E-24	0.08
Atp9a	1.99E-04	5.22E-03	0.15
Etnk2	8.96E-03	9.95E-02	2.78
Sphingolipid metabolism			
	P Value	FDR	FC
Sphk1	1.59E-03	2.70E-02	3.09
Structural lipoprotein			
	P Value	FDR	FC
Apol11a	1.08E-15	3.18E-13	0.02
Apol10a	2.46E-09	2.89E-07	0.07
Apol10c-ps	3.92E-04	9.01E-03	28.10
Transcription factor			
	P Value	FDR	FC
Rxra	1.45E-08	1.49E-06	2.63
Cebpa	6.21E-05	2.03E-03	5.84
Ppargc1a	7.83E-04	1.57E-02	8.81
Nr1h3	8.02E-03	9.20E-02	2.47
Pparg	3.86E-05	1.39E-03	5.41
Unfolded protein response			
	P Value	FDR	FC
Map3k5	2.44E-11	4.20E-09	22.85
Creb3l1	1.77E-03	2.93E-02	0.38
Creb3	4.32E-03	5.83E-02	0.41
Autophagy			
	P Value	FDR	FC
Rb1cc1	2.02E-04	5.30E-03	0.43

Table S2. Effect of oncogenic APC on cellular gene expression. Differentially expressed genes associated with lipid metabolism, cytoskeleton and other metabolic pathways in **A)** IMCE (Apc 850/+) and **B)** IMCE βcat (Apc 850/+ βcat -/+) cultured colonocytes. IMCE and IMCE βcat cells were compared to isogenic YAMC ($Apc^{+/+}$) cells. Approximately 350 genes were differentially expressed using an FDR<0.1 cutoff value, *n*=3 biological replicates per cell line. Genes are grouped according to their role in cellular processes. Cellular processes are listed in alphabetical order and genes within each group are ordered from smallest to largest FDR. FC, fold change expressed as IMCE or IMCE βcat / YAMC. Source data are provided as a Source Data file.
