# Supplementary material for: Mutant APC reshapes Wnt signaling plasma membrane nanodomains by altering cholesterol levels via oncogenic β-catenin
Source: Nat Commun. 2023 Jul 19;14:4342. doi: 10.1038/s41467-023-39640-w (PMC10356786; doi:10.1038/s41467-023-39640-w)
Supplement: Supplementary file 3 — Reporting Summary [file 41467_2023_39640_MOESM3_ESM.pdf]

nature portfolio

Corresponding author(s): Robert S. Chapkin

Last updated by author(s): 06/10/2023

Reporting Summary

Nature Portfolio wishes to improve the reproducibility of the work that we publish. This form provides structure for consistency and transparency in reporting. For further information on Nature Portfolio policies, see our Editorial Policies and the Editorial Policy Checklist.

Statistics

For all statistical analyses, confirm that the following items are present in the figure legend, table legend, main text, or Methods section.

n/a	Confirmed	
☐	☒	The exact sample size (*n*) for each experimental group/condition, given as a discrete number and unit of measurement
☐	☒	A statement on whether measurements were taken from distinct samples or whether the same sample was measured repeatedly
☐	☒	The statistical test(s) used AND whether they are one- or two-sided<br>*Only common tests should be described solely by name; describe more complex techniques in the Methods section.*
☐	☒	A description of all covariates tested
☐	☒	A description of any assumptions or corrections, such as tests of normality and adjustment for multiple comparisons
☐	☒	A full description of the statistical parameters including central tendency (e.g. means) or other basic estimates (e.g. regression coefficient) AND variation (e.g. standard deviation) or associated estimates of uncertainty (e.g. confidence intervals)
☐	☒	For null hypothesis testing, the test statistic (e.g. *F*, *t*, *r*) with confidence intervals, effect sizes, degrees of freedom and *P* value noted<br>*Give P values as exact values whenever suitable.*
☒	☐	For Bayesian analysis, information on the choice of priors and Markov chain Monte Carlo settings
☒	☐	For hierarchical and complex designs, identification of the appropriate level for tests and full reporting of outcomes
☒	☐	Estimates of effect sizes (e.g. Cohen's *d*, Pearson's *r*), indicating how they were calculated

Our web collection on statistics for biologists contains articles on many of the points above.

Software and code

Policy information about availability of computer code

Data collection	GP and fluorescence intensity: LAS X 3.5.7.23225 , Leica; NIS-Elements AR 5.41.02 and Inspire for FlowSight 200.1.680.0, Amnis;<br><br>FLIM-FRET: LI-FLIM 1.2.26, Lambert Instruments BV;<br><br>Super resolution TIRF-STORM: NIS elements AR ProEM-nSTORM 5.41.02, Nikon;<br><br>Sorted cells: ProSort 1.6, Bio-Rad;<br><br>Chemiluminescence: CLARIOstar Plus Control, BMG Labtech;<br><br>Western blot and DNA gel imaging: ImageLab Touch 2.3.0.07, Bio-Rad;<br><br>RNA expression: EdgeR 3.42.4, Bioconductor and<br><br>Cancer genomics: cBioPortal, Memorial Sloan Kettering Cancer Center
Data analysis	GP and fluorescence analysis and Super resolution TIRF-STORM protein cluster particle analysis: Image J "Fiji"2.9.0/1.53t, open source;<br><br>Super resolution TIRF-STORM protein cluster particle analysis: MATLAB 9.13.0.2193358, MathWorks<br><br>FLIM-FRET: LI-FLIM 1.2.26, Lambert Instruments BV;

Flow cytometry analysis: IDEAS 6.2, Amnis;

Sorted cells ProSort 1.6, Bio-Rad;

Chemiluminescence: MARS 4.2, BMG Labtech;

Western blot and DNA gel imaging: ImageLab Touch 2.3.0.07, Bio-Rad;

RNA expression: GSEA 4.1.0, UCSD-Broad Institute and

Cancer genomics: cBioPortal, Memorial Sloan Kettering Cancer Center

For manuscripts utilizing custom algorithms or software that are central to the research but not yet described in published literature, software must be made available to editors and reviewers. We strongly encourage code deposition in a community repository (e.g. GitHub). See the Nature Portfolio guidelines for submitting code & software for further information.

Data

Policy information about availability of data

All manuscripts must include a data availability statement. This statement should provide the following information, where applicable:

- Accession codes, unique identifiers, or web links for publicly available datasets
- A description of any restrictions on data availability
- For clinical datasets or third party data, please ensure that the statement adheres to our policy

Bowel-associated CRC dataset: https://www.cbioportal.org

RNAseq data have been deposited in the Gene Expression Omnibus (GEO) database (accession number is pending)

Code/macros have been deposited in The Chapkin Lab public depository: https://github.com/chapkinlab/Mutant-APC-reshapes-plasma-membrane-Nat-Comms.git

Source codes for the underlying functions and graphical user interface (GUI) application ClusDoc are available at the authors' Git repository (https://github.com/PRNicovich/ClusDoC).

Human research participants

Policy information about studies involving human research participants and Sex and Gender in Research.

Reporting on sex and gender	not applicable
Population characteristics	not applicable
Recruitment	not applicable
Ethics oversight	not applicable

Note that full information on the approval of the study protocol must also be provided in the manuscript.

Field-specific reporting

Please select the one below that is the best fit for your research. If you are not sure, read the appropriate sections before making your selection.

☒ Life sciences ☐ Behavioural & social sciences ☐ Ecological, evolutionary & environmental sciences

For a reference copy of the document with all sections, see nature.com/documents/nr-reporting-summary-flat.pdf

Life sciences study design

All studies must disclose on these points even when the disclosure is negative.

Sample size	Sample size was determined based of previous published in cellulo and animal studies perform in our lab (PMID: 29769200, 33515553, 31630812), which provided information associated with standard deviation, type 1 error, power, direction of effect, statistical tests and expected attrition. For animal studies, sample size was further confirmed employing a method based on law of diminishing return. This method is called "resource equation" (PMID: 24250214). The final sample size value was adjusted by taking into consideration the expected attrition.
Data exclusions	No data were excluded from the analyses.

Replication	The experimental findings are the result of independent biological experiments perform in replicates (n). In most scenarios/cases, independent experiments were performed on different days.
Randomization	Organisms were randomized into groups via the block randomization method (PMID: 21772732)
Blinding	Investigators were blinded during data collection and analysis. In vitro/in cellulo samples were assigned numbers randomly and organisms an identification number (ID) given at birth. These identifiers were recorded and matched to the sample or organism post data collection and analysis.

Reporting for specific materials, systems and methods

We require information from authors about some types of materials, experimental systems and methods used in many studies. Here, indicate whether each material, system or method listed is relevant to your study. If you are not sure if a list item applies to your research, read the appropriate section before selecting a response.

Materials & experimental systems

n/a	Involved in the study
☐	☒ Antibodies
☐	☒ Eukaryotic cell lines
☒	☐ Palaeontology and archaeology
☐	☒ Animals and other organisms
☒	☐ Clinical data
☒	☐ Dual use research of concern

Methods

n/a	Involved in the study
☒	☐ ChIP-seq
☐	☒ Flow cytometry
☒	☐ MRI-based neuroimaging

Antibodies

Antibodies used

1. anti-LRP6; R & D Systems; MAB2960; Clone # 290913
2. anti-Frizzled7; R & D Systems; MAB1981-100; Clone # 151143
3. anti-rat CF568; Biotium; 20092
4. anti-rat AF647; Invitrogen; A21247
5. anti-Lacz; Developmental Studies Hybridoma Bank; 40-1a
6. anti-APC for human colonocytes; Abcam; ab40778
7. anti-APC for mouse colonocytes; EMD Millipore; OP44
8. anti-Abca1; Cell Signaling; 96292
9. anti-Abcg1; Abcam; b52617
10. anti-Lrp8; Invitrogen; PA1-16913
11. anti-Scarf1; Invitrogen; PA5-115870
12. anti-βcat; BD Biosciences; 610154
13. anti-GAPDH; Santa Cruz Biotechnology; sc-365062
14. anti-β-actin; Cell Signaling; 4970S
15. anti-rabbit HRP; Seracare; 5220-0480
16. anti-mouse; Seracare; 5450-0011
17. anti-Fzd7-AF647; R&D Systems; FAM1981R
18. anti-LRP6-AF647; R&D Systems; FAB1505R
19. anti-Dvl1-AF647; Santa Cruz Biotechnology; sc-8025 AF647
20. Donkey Alexa Fluor® 488 AffiniPure Donkey Anti-Mouse; Jackson Immunoresearch; 715-545-150
21. Cy™3 AffiniPure Donkey Anti-Mouse IgG; Jackson Immunoresearch; 715-165-150

22. Donkey anti mouse Alexa Fluor 647; Jackson Immunoresearch; 715-605-150

Validation	1. LRP6 Antibody (PMID: 23022962)<br>2. Frizzled-7 Antibody (PMID: 32283254, 7546602, 19265664, 22536330)<br>3. anti-Lacz Antibody (PMID: 1658618, 31409797, 32194035, 33556132)<br>4. anti-APC 1 (PMID: 35654814, 35320710, 33454586, 34946869, 34267342)<br>5. anti-APC 2 (PMID: 8373591, 8385345, 8389242, 1565631, 1348017)<br>6. anti-Abca1 (PMID: 24081377, 12763760, 10431238, 10431237, 10431236)<br>7. anti-Abcg1 (PMID: 33951300, 33404706, 33996254, 33490072, 33330456)<br>8. anti-βcat (PMID: 10629227, 23085754, 12235124, 2349235)<br>9. anti-Fzd7-AF647 (PMID: 17884187, 17485397, 9813155, 10727861, 17576136, 19497282, 18681827, 17016432)

Eukaryotic cell lines

Policy information about cell lines and Sex and Gender in Research

Cell line source(s)	YAMC and IMCE cell lines were were kindly provided by R.H. Whitehead of the Ludwig Cancer Institute (Melbourne, Australia). The IMCE Beat cell line was kindly provided by L.M. Matrisian of the Department of Cancer Biology, Vanderbilt University School of Medicine (Nashville, Tennessee). Leading Light Wnt Reporter 3T3 mouse fibroblasts (3T3) were obtained from Enzo Life Sciences (Farmingdale, NY). CRC cell lines, i.e., HCT116, SW480, DLD1 and HT29, were obtained from ATCC (Manassas, VA). HCT116Δ was obtained from Horizon Discovery (Waterbeach, UK). HAP1 parental and KO cell lines were obtained from Horizon Discovery (Waterbeach, UK).
Authentication	YAMC, IMCE and IMCE Bcat cell lines were authenticated by STR profiling (CellCheck Plus) by IDEXX BioResearch (Westbrook, Maine). All other cell lines were authenticated by the corresponding vendor.
Mycoplasma contamination	All cell lines employed in our experiments tested negative for mycoplasma bacteria as assessed by a universal mycoplasma detection kit (30-1012K; American Type Culture Collection, Manassas (ATCC), VA)
Commonly misidentified lines<br>(See ICLAC register)	No commonly misidentified cell lines were used in our studies.

Animals and other research organisms

Policy information about studies involving animals; ARRIVE guidelines recommended for reporting animal research, and Sex and Gender in Research

Laboratory animals	C57BL/6 mice were used in our studies. Mice weaning was performed at 8 weeks of age. All mice in these studies were between 4-6 months of age (16-24 weeks). Mice were housed in cages in a temperature between 68°F - 79°F (set point 73°F) and humidity between 50%-70% controlled animal facility with a 12 h light/dark cycle and fed standard chow.<br><br>All flies were backcrossed ×10 into the w1118 background, with continued backcrossing every 6–8 months to maintain isogenecity. All flies were reared on standard yeast- and cornmeal-based diet at 25° C and 65% humidity on a 12 h light/dark cycle. Crosses and flies were kept at 18° C (permissive temperature), and 5-day-old females were fed the experimental cholesterol diets for 5 days, and then shifted to 29° C for 2 days to allow for expression of the transgenes before analysis. For experiments utilizing the transgenic Wnt reporter, crosses and flies were kept at 18° C (permissive temperature) and 5-day-old females were fed the experimental cholesterol diets for 2 days, and subsequently shifted to 29° C for 5 days to allow for expression of the transgenes before analysis.
Wild animals	Our studies did not involve wild animals.
Reporting on sex	Our findings do not depend on the sex of the animal. An even number of males and females were assigned to each condition group for all animal experiments.
Field-collected samples	Our study did not involve field-collected samples.
Ethics oversight	All experiments utilizing mice were approved and conducted in rigorous accordance with the Texas A&M University Institutional Animal Care and Use Committee and conformed to NIH guidelines. Furthermore, all personnel have satisfactorily completed the CITI Biomedical Responsible Conduct of Research Training.

Note that full information on the approval of the study protocol must also be provided in the manuscript.

Flow Cytometry

Plots

Confirm that:

☒ The axis labels state the marker and fluorochrome used (e.g. CD4-FITC).

☒ The axis scales are clearly visible. Include numbers along axes only for bottom left plot of group (a 'group' is an analysis of identical markers).

☒ All plots are contour plots with outliers or pseudocolor plots.

☒ A numerical value for number of cells or percentage (with statistics) is provided.

Methodology

Sample preparation	Sample preparation was performed using previously established laboratory protocols. The details have been provided and described in detail in the Materials and Methods section
Instrument	1. Luminex Amnis FlowSight<br><br>2. Bio-Rad S3e Cell Sorter
Software	1. Luminex Amnis FlowSight<br>Collection Software: Inspire for FlowSight 200.1.680.0<br>Analysis Software: IDEAS 6.2<br><br>2. Bio-Rad S3e Cell Sorter<br>Collection Software: ProSort<br>Analysis Software: ProSort
Cell population abundance	For colonic stem cell experiments, only Lgr5+ (GFPhigh) cells were collected via FACS. Lgr5+ cells range between ~8-22% of the total cell population depending on the condition tested as shown in Supplementary data. Sorting purity was routinely examined and over 95%.<br><br>For other flow cytometry studies, no sorting was performed and the entire/whole cell population was analyzed.<br>Relevant citations: (PMID: 32915464, 33495399, 27831561, 22750333).
Gating strategy	Gating strategies were previously established using published in-house protocols (PMID: 32915464, 33495399, 27831561, 22750333).

☒ Tick this box to confirm that a figure exemplifying the gating strategy is provided in the Supplementary Information.
